# The dark side of the moon: first insights into the microbiome structure and function of one of the last glacier-fed streams in Africa

**DOI:** 10.1098/rsos.230329

**Published:** 2023-08-09

**Authors:** Grégoire Michoud, Tyler J. Kohler, Leïla Ezzat, Hannes Peter, Juliet Kigongo Nattabi, Rosemary Nalwanga, Paraskevi Pramateftaki, Michail Styllas, Matteo Tolosano, Vincent De Staercke, Martina Schön, Ramona Marasco, Daniele Daffonchio, Massimo Bourquin, Susheel Bhanu Busi, Tom J. Battin

**Affiliations:** ^1^ River Ecosystems Laboratory, Alpine and Polar Environmental Research Center, Ecole Polytechnique Fédérale de Lausanne (EPFL), Lausanne, Switzerland; ^2^ Department of Zoology, Entomology and Fisheries Sciences (ZEFs), College of Natural Sciences, Makerere University, Kampala, Uganda; ^3^ Systems Ecology Group, Luxembourg Centre for Systems Biomedicine, University of Luxembourg, Esch-sur-Alzette, Luxembourg; ^4^ Biological and Environmental Sciences and Engineering Division (BESE), King Abdullah University of Science and Technology (KAUST), Thuwal, Saudi Arabia

**Keywords:** microbial ecology, biofilms, tropical glacier, Africa, climate change, Uganda

## Abstract

The glaciers on Africa's ‘Mountains of the Moon' (Rwenzori National Park, Uganda) are predicted to disappear within the next decades owing to climate change. Consequently, the glacier-fed streams (GFSs) that drain them will vanish, along with their resident microbial communities. Despite the relevance of microbial communities for performing ecosystem processes in equatorial GFSs, their ecology remains understudied. Here, we show that the benthic microbiome from the Mt. Stanley GFS is distinct at several levels from other GFSs. Specifically, several novel taxa were present, and usually common groups such as Chrysophytes and *Polaromonas* exhibited lower relative abundances compared to higher-latitude GFSs, while cyanobacteria and diatoms were more abundant. The rich primary producer community in this GFS likely results from the greater environmental stability of the Afrotropics, and accordingly, heterotrophic processes dominated in the bacterial community. Metagenomics revealed that almost all prokaryotes in the Mt. Stanley GFS are capable of organic carbon oxidation, while greater than 80% have the potential for fermentation and acetate oxidation. Our findings suggest a close coupling between photoautotrophs and other microbes in this GFS, and provide a glimpse into the future for high-latitude GFSs globally where primary production is projected to increase with ongoing glacier shrinkage.

## 1. Background

Glaciers are shrinking worldwide owing to climate change [1,2]. Nowhere is this change more conspicuous than in the Earth's tropical regions (e.g. equatorial South America, Africa and Papua New Guinea), where few even realize glaciers exist. Given that many tropical glaciers are predicted to disappear within years or decades [3,4], it was recently suggested that we might be among the last generations to explore the ecology of these unique and endangered ecosystems [5], creating an urgent need to investigate them.

In Africa, a few glaciers still coronate the peaks of Mt. Kilimanjaro, Mt. Kenya and the Rwenzori Mountains [6–8]. The latter of these, the legendary ‘Mountains of the Moon', famously described by the ancient Greek scholar Ptolemy [9], are also the least explored in Africa. The Rwenzori Mountains have long been heralded as an important area for biodiversity, particularly endemic species [9], which is one of the reasons for its coveted UNESCO World Heritage status (https://whc.unesco.org/en/list/684/). The glaciers of the Rwenzori Mountains have long served as a headwater source to iconic lakes [10,11], as well as the White Nile [9]. Yet, ice volume has diminished drastically over the last decades, and the glaciers are predicted to disappear entirely within the next few decades [3,12].

As mountain glaciers shrink worldwide, the streams they feed (glacier-fed streams; GFSs) are vanishing. In temperate and polar regions, GFSs are typically characterized by pronounced daily and seasonal shifts in the runoff, and concomitantly by marked fluctuations in temperature, suspended sediment loads and light availability [13]. Alongside unstable channels, the environment of these GFSs is generally unfavourable to primary production, except during the vernal and autumnal windows of opportunity [14,15]. Tropical GFSs differ from this template: their hydrological regime does not display pronounced seasonal patterns and diurnal fluctuations remain small [16,17]. This regime translates into greater hydrological and channel stabilities, likely improving the habitability of tropical GFS ecosystems, particularly for primary producers.

Here, we provide a first look into the microbiome structure and function of benthic biofilms inhabiting an African GFS (Mt. Stanley, Rwenzori Mountains). Understanding biofilm structure and function is critical because biofilms regulate critical ecosystem processes in streams and rivers with consequences for large-scale biogeochemical fluxes [13]. Performing 16S and 18S rRNA gene amplicon sequencing, we evaluated the microbial community structure of epilithic (i.e. growing on boulders) and epipsammic (i.e. growing on sand grains) biofilms. Furthermore, employing genome-centric metagenomics, we investigated potential energy acquisition pathways and metabolic traits of the microbial communities from these two habitat types. Our study represents a major contribution to our knowledge of the tropical cryospheric microbiome, which is largely restricted to cryoconite communities and ice algae to date [18–20]. Our findings also highlight the autotrophic character of the Mt. Stanley GFS, and hence provide a look into the future of GFSs at higher latitudes which are predicted to develop into ‘greener’ ecosystems as their glaciers shrink [14,21]

## 2. Material and methods

### 2.1. Site description and sample collection

**Figure 1.**
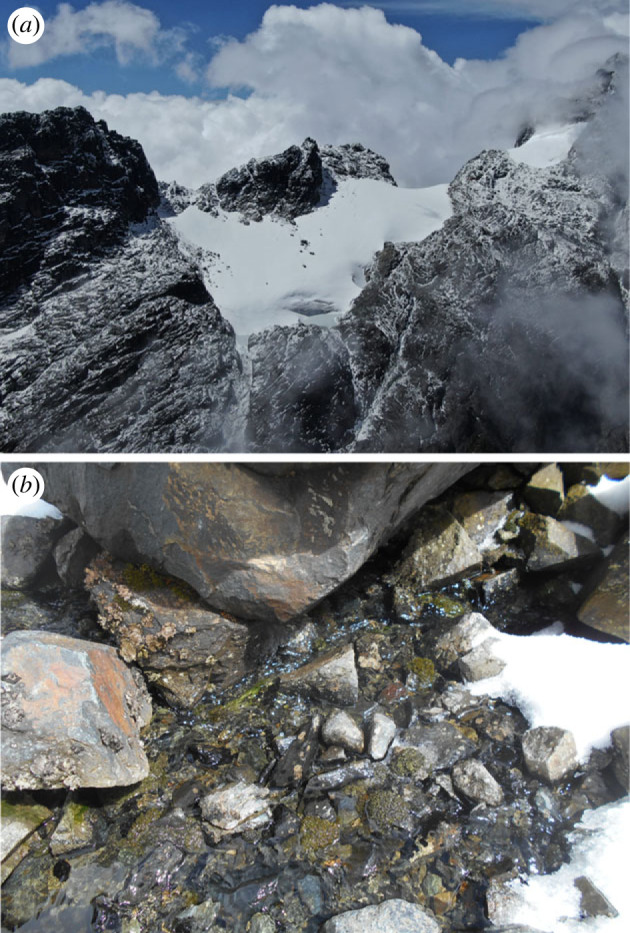
Photographs showing the Mt. Stanley Glacier and its stream channel to the bottom left (*a*), and the bottom of the Mt. Stanley glacier-fed stream with angular sediments, green algae and mosses (*b*).

The glaciers of the Rwenzori Mountains have been receding since at least the second half of the nineteenth century [22], with remnants still present on Mts. Speke, Baker and Stanley. The latter of these is the third highest peak in Africa, with the Mt. Stanley Glacier estimated to be 2.85 km^2^ in area at the beginning of the twentieth century, and reduced to approximately 0.5 km^2^ at the beginning of the twenty-first century [3]. At the time of our expedition (December 2021), the glacier area was estimated at 0.04 km^2^ using satellite imagery, and the glacier snout (N 00.3757, E 29.8781) was located at 4742 m above sea level (figure 1*a*). The Rwenzori Mountains are comprised primarily of uplifted schists and gneisses [9,23], and the streambed of the Mt. Stanley GFS is characterized by angular cobbles and boulders interspersed by sandy sediments. Here, well-developed patches of mosses and algae were present, indicative of an armoured and stable streambed (figure 1*b*).

We sampled two reaches of the Mt. Stanley GFS located at 43 and 86 m downstream from the glacier snout (the upper (UP) and lower (DN) transects, electronic supplementary material, table S1). In both reaches, we collected three benthic (less than 5 cm depth) sandy sediment samples for epipsammic biofilms using flame-sterilized metal scoops and sieves (0.25–3.15 mm mesh size, six samples total). Six epilithic biofilms were also collected using a sterilized metal spatula from larger boulders (i.e. three boulders from each of the two reaches). All samples were flash-frozen *in situ* with liquid nitrogen and kept frozen (−80°C) pending analyses. Both epipsammic and epilithic biofilms subsequently underwent DNA extraction, and epipsammic samples only were further analysed for microbial biomass and extracellular enzyme activity (EEA).

### 2.2. Streamwater physico-chemical characteristics

In both reaches, we measured streamwater pH, electrical conductivity (EC) and O_2_ concentration (MultiLine Multi 3630 IDS, WTW, Germany), as well as turbidity (Turb 430 IR, WTW). Streamwater samples for nutrient analyses were filtered (pre-combusted Whatman GF/F filters) into acid-washed Nalgene HDPE bottles and frozen (−20°C). Concentrations of streamwater nitrate (${\mathrm{NO}}_{2}^{-}+{\mathrm{NO}}_{3}^{-}$; QuikChem Method 10-107-05-1-C), nitrite + nitrate (${\mathrm{NO}}_{3}$; 10-107-04-1-B), ammonium (NH_4_^+^; 10-107-06-3-D) and soluble reactive phosphorus (SRP; 10-115-01-1-M) were analysed using a LaCHat QuikChem 8500 flow injection analyser. Dissolved organic carbon (DOC) samples from the upstream reach only were filtered (pre-combusted Whatman GF/F filters) into acid-washed amber glass vials and stored (4°C) until analysis on a Sievers M9 TOC Analyser (GE, Boston, MA, USA). Streamwater samples for the analysis of major cations and anions were sterile-filtered (0.2 µm), stored (4°C), and analysed on a Metrohm 930 Compact IC flex system.

### 2.3. Epipsammic chlorophyll-a and extracellular enzyme activities

Chlorophyll-*a* and EEA associated with the epipsammic biofilms were quantified as previously described [21]. Briefly, we extracted chlorophyll-*a* from each sample in triplicate using 90% EtOH in a hot water bath (78°C, 10 min) followed by 24 h incubation (4°C in the dark); after centrifugation, the supernatant was read for chlorophyll-*a* on a plate reader (BioTek Synergy H1 high sensitivity, excitation 436 nm; emission: 680 nm).

We quantified the potential activities of *α*-1,4-glucosidase, β-1,4-glucosidase, leucine aminopeptidase, β-1,4-*N*-acetylglucosaminidase and acid (alkaline) phosphatase using fluorescent 4-Methylumbelliferone (MUF) and 7-Amino-4-methylcoumarin (AMC)-linked substrates. Substrates were dissolved in artificial streamwater (pH = 7.5) at a concentration of 0.3 µM, added to the sediment sample (*ca* 1 g for each sample, analysed in triplicates) and incubated on a shaker for 1.5–2 h (4°C, in the dark); the reaction was stopped with glycine buffer (pH = 10.4). Samples were then vortexed, centrifuged and read on a BioTek Synergy H1 high sensitivity plate reader at 365/455 excitation/emission wavelengths for MUF and 364/445 excitation/emission wavelengths for AMC. EEAs were calculated and normalized to dry mass sediment as done previously [24].

### 2.4. Nucleic acid extraction and amplicon sequencing

Nucleic acids were extracted from the six epipsammic and six epilithic biofilm samples using a modified phenol-chloroform protocol [25]. Prokaryotic community composition was characterized by amplifying the V3–V4 hypervariable region of the 16S rRNA gene using primers 341f (5′-CCTACGGGNGGCWGCAG-3′) and 785r (5′-GACTACHVGGGTATCTAATCC-3′; [26]). For eukaryotes, the V4 loop of the 18S rRNA gene was amplified using the TAReuk454F––TAReukREV3 primers [27]. PCR amplification was performed with the KAPA HiFi DNA polymerase in a Biometra Trio (Biometra) in 25 cycles. Amplicon libraries were prepared according to the MiSeq manufacturer's protocol, and samples were sequenced using a 300 bp paired-end protocol on the Illumina MiSeq platform in the Bioscience Core Lab of King Abdullah University of Science and Technology in Saudi Arabia.

### 2.5. Quality control of 16S and 18S rRNA gene reads, and initial data processing and data analyses

A total of 12 16S and 18S rRNA gene amplicon libraries were generated, which included six epipsammic and six epilithic samples for each primer pair. Paired-end sequencing produced 2 077 151 and 2 256 066 reads for the 16S and 18S rRNA gene datasets, respectively, including 173 095 and 188 005 average reads. Amplicon data were trimmed using Trimmomatic (v.0.36, [28]). Briefly, reads were truncated in 4 bp sliding windows when mean quality dropped below a Phred score of 15. We removed the three leading and trailing nucleotides and discarded reads shorter than 200 bp. We then used the Quantitative Insights into Microbial Ecology 2 (QIIME2, v.2019.1, [29]) workflow for sequence processing. Amplicon sequence variants (ASVs) were created using DADA2 [30]. Briefly, 17 and 21 nucleotides were discarded at the beginning of each forward and reverse read, and forward and reverse reads were truncated at 280 and 220 bases for the 16S rRNA gene dataset. For the 18S rRNA gene dataset, 18 and 20 nucleotides were discarded at the beginning of each forward and reverse read, and forward and reverse reads were truncated at 280 and 200 bases. These different steps resulted in 1 313 277 and 1 830 970 reads for 16S and 18S rRNA gene datasets, respectively. The SILVA database (v138.1) was then used for taxonomic classification [31]. Singletons, doubletons, mitochondria, chloroplasts and unknown phyla were removed prior to performing analyses of the 16S rRNA gene dataset. Singletons, doubletons, non-eukaryotic, Conoidasida-related sequences were discarded from the 18S rRNA dataset. These last steps resulted in 1 214 913 and 1 801 286 reads for the 16S and 18S rRNA gene datasets, respectively. The data were rarefied with rarefaction thresholds set at 78 745 and 86 058 for the two datasets, respectively. All analyses were performed in R (v.4.1) [32]. Samples from both reaches were combined together for further analyses. To assess differences in the composition of bacterial and eukaryotic communities between epipsammic and epilithic samples, we computed PERMANOVA analyses using the function ‘adonis2' implemented in the package *vegan* [33]. Furthermore, we took advantage of the available 16S and 18S rRNA gene amplicon sequence datasets from the Vanishing Glaciers Project (https://www.glacierstreams.ch/) to compare the Mt. Stanley GFS communities with corresponding (i) GFS epipsammic biofilms from the Southern Alps, Southwest Greenland, the Scandinavian Mountains, the European Alps, the Ecuadorian Andes and the Caucasus Mountains [21], and (ii) epilithic biofilms from the Caucasus Mountains and the Southern Alps [34], respectively. We note that these studies used the same primer pairs, allowing us to reduce potential biases for the statistical comparisons. For these, we computed PERMANOVA analyses (adonis2) and tested for the effect of the ‘mountain range' on both bacterial (16S) and eukaryote (18S) community structure. Pairwise differences were assessed with the function ‘pairwise.adonis' in the package *pairwiseAdonis* (v.0.4) [35], including a false discovery rate correction to account for multiple comparisons. Differential abundance of taxa between the Mt. Stanley samples versus the already published GFS epipsammic biofilms in different regions and between episammic and epilithic biofilms in Mt Stanley samples were performed with ANCOMBC (v1.4) in R [36]. We considered significantly enriched taxa, the ones with a *W* statistic above the threshold (0.7) and an adjusted *p*-value < 0.01.

### 2.6. Metagenomic sequencing, assembly and selection of genomes

Twelve shotgun metagenomics libraries were prepared using the NEBNext Ultra II FS library kit as described previously [34]. Briefly, 50 ng of DNA was enzymatically fragmented (12.5 min), followed by six PCR amplification cycles for constructing the libraries. The libraries were quantified using Qubit (Invitrogen), and the average insert size (approx. 450 bp) was determined by quality assessment using the Bioanalyzer from Agilent. All libraries were pooled and sequenced at the Functional Genomics Centre Zurich on a NovaSeq (Illumina) using an S4 flowcell (2 × 150 bp).

To determine microbiome diversity and community structure, SingleM (v1.0.0beta5) was applied to reads from each sample [37].

The metagenomic assembly was performed individually per sample. First, the reads were trimmed and quality controlled with trimmomatic [28] to remove adapter sequences and leading and trailing bases with a quality score below 20 and reads with an average of 20 per base quality over a 4-bp window. Then, preprocessed reads were assembled using Megahit (v1.2.9) with a minimum contig length of 1000 bp and default parameters [38], all done using the Integrated Meta-omic Pipeline (IMP) workflow (v3.0.0) [39] (electronic supplementary material, table S2). Using BWA (v 0.7.17) (mem option) [40], we mapped all reads with each assembly individually to obtain coverage information from multiple samples. Each metagenome was binned individually using: MetaBat2 (v2.1.12), Concoct (v1.1.0) and Metabinner (v1.4.3) using default parameters and a minimal contig length of 1500 bp expect for Metabinner where the minimum length was 2000 bp [41–43]. We consolidated the bins using DASTool (v1.1.4) with default settings [44], resulting in a total of 2350 bins. Next, we dereplicated these in metagenome-assembled genomes (MAGs) using dRep (v2.3.2) [45] with a minimum completeness and maximum contamination of 70% and 10%, respectively, as determined by CheckM (v1.2.0) [46] and a total of 682 prokaryotic MAGs were obtained. CheckM and GTDB-Tk (v2.1.0) were used, respectively, to determine the quality and taxonomy of MAGs [47] (https://github.com/michoug/UgandaAnalysis). Only those considered medium quality or greater were considered (i.e. completeness above 70% and contamination less than 10%) [48] (electronic supplementary material, table S3). The MAGs were functionally annotated with Bakta (v1.1), EggNog Mapper (v2.1.8) and METABOLIC (v4.0) using default settings [49–51]. We used fastANI (v1.33) [52] to evaluate the uniqueness of the datasets by estimating if MAGs belonged to the same species as other publicly available ones in similar environments such as GFSs and glaciers [34,53]. CoverM (v0.6.1) [54] with BWA was used to determine the coverage of the reference MAGs with the rpkm method. The coverage per sample was normalized after mapping the reads to the *recA* gene obtained from the assemblies.

## 3. Results and discussion

### 3.1. Resources and microbial activities in the Mt. Stanley GFS

Streamwater temperature, specific conductivity and major ion concentrations were overall low, which may be partially attributable to snowmelt (electronic supplementary material, table S1 and S4); however, solute concentrations are within the range of other GFSs, including those from temperate [55] and polar regions [56]. Among the nitrogen (N) species, nitrate concentration was highest, followed by ammonium and nitrite, whereas soluble reactive phosphorus (P) was slightly above detection. As a result, the average molar N:P ratio was 485.8, reflecting the disproportionate prevalence of N over P in the Mt. Stanley GFS. The average DOC concentration was relatively high compared to other high-altitude [21] and high-latitude GFSs [56], leading to molar C:P and C:N ratios of 1438 and 3, respectively. Thus, while P is limiting with respect to C and N, C is likely also to be limiting with respect to N, assuming a Redfield ratio of 106:16:1 (C:N:P [57]).

Overall, the biomass of epipsammic algae was relatively high in the Mt. Stanley GFS and increased downstream as indicated by increasing chlorophyll-*a* content (from 0.38 ± 0.16 to 1.63 ± 0.03 µg g^−1^ DM sediment, mean ± s.d.). To put these values into context, the overall mean, median and range of chlorophyll-*a* concentration from the 101 GFSs reported in Kohler *et al*. [21] were 0.023, 0.004 and 0–0.511 µg g^−1^ DM sediment, respectively, and the lowest individual observation from the Mt. Stanley GFS was greater than 97% of the observations in that dataset. We attribute this relatively high biomass to the channel stability, little erosive forces of the moderate water flow, and high light availability because of low turbidity (less than 10 NTU, electronic supplementary material, table S1). The wide occurrence of mosses on the streambed underpins this notion of environmental stability, even on a longer timescale. Elevated autotrophic biomass, in combination with relatively low discharge, may cause the relatively high DOC concentration in the Mt. Stanley GFS (474 ppb, electronic supplementary material, table S4). Benthic algae are well known to exude polysaccharides from excess photosynthesis that contribute to the streamwater DOC pool [58]. At the same time, we suggest that this high primary production is one possible cause of the low P concentration in the Mt. Stanley GFS, coupled with relatively low inputs from subglacial and streambed weathering [59].

Furthermore, the benthic autotrophic biomass may serve as an important organic carbon source for microbial heterotrophs in the Mt. Stanley GFS biofilms. We thus measured extracellular enzymatic activities (EEAs) to infer elemental acquisition effort related to C (α- and β-1,4-glucosidase), N (leucine aminopeptidase and *N*-acetylglucosaminidase) and P (phosphatase) [60]. In general, measured EEA values were high relative to other GFSs globally (table 1). For example, the total average EEA from the Mt. Stanley GFS (265.12 nmol g^−1^ DM h^−1^) was 54 times higher than the average total EEA from 101 GFSs around the globe (4.90 nmol g^−1^ DM h^−1^; [21]) indicating a greater level of heterotrophic activity in this stream likely due to elevated photoautotrophy. The activity of β-glucosidase is particularly noteworthy, as it exhibited the greatest activity of the five EEA measured for the Mt. Stanley GFS. Interestingly, leucine aminopeptidase, which degrades proteins to acquire N (as well as C), typically exhibits the highest activity in GFSs; across 101 GFSs globally, leucine aminopeptidase (3.06 nmol g^−1^ DM h^−1^) had higher average activities than β-glucosidase (0.31 nmol g^−1^ DM h^−1^) by approximately one order of magnitude [21,24]. Yet in the Mt. Stanley GFS, the average β-glucosidase activity (142.31 nmol g^−1^) was almost one order of magnitude higher than leucine aminopeptidase activity (15.14 nmol g^−1^ DM h^−1^). β-glucosidase primarily cleaves cellulose, which is an important structural component of chlorophytes. Therefore, it seems that the microbial heterotrophs in the Mt. Stanley GFS benefit from carbon sources provided by algae. In agreement with this, the higher algal biomass observed in the downstream reach was also reflected by elevated EEA (table 1). A further reason for the lower leucine aminopeptidase activity may be the high N:P ratio of streamwater nutrients. Given the relative abundance of streamwater N over P, communities may therefore invest less energy in the synthesis of N-acquiring enzymes (i.e. leucine aminopeptidase and *N*-acetylglucosaminidase) but more into *p*-acquiring enzymes (i.e. phosphatase). Indeed, we found phosphatase in the Mt. Stanley GFS to have approximately twice the activity of leucine aminopeptidase and *N*-acetylglucosaminidase combined (table 1).

**Table 1.** Measured chlorophyll-*a* (Chla) concentrations and extracellular enzyme activity (EEA) rates of benthic sediments. Measured EEA include: α-1,4-glucosidase, acid (alkaline) phosphatase, β-1,4-glucosidase, leucine aminopeptidase and β-1,4-*N*-acetylglucosaminidase. Values given are the mean ± standard deviation of six patches.

Chla (µg g^−1^)	α-glucosidase (nmol g^−1^ h^−1^)	phosphatase (nmol g^−1^ h^−1^)	β-glucosidase (nmol g^−1^ h^−1^)	leucine aminopeptidase (nmol g^−1^ h^−1^)	*N*-acetylglucosaminidase (nmol g^−1^ h^−1^)
1.01 ± 0.69	5.84 ± 8.17	77.74 ± 57.94	142.31 ± 206.37	15.14 ± 10.75	24.09 ± 22.54

### 3.2. Microbiome structure of the Mt. Stanley GFS

**Figure 2.**
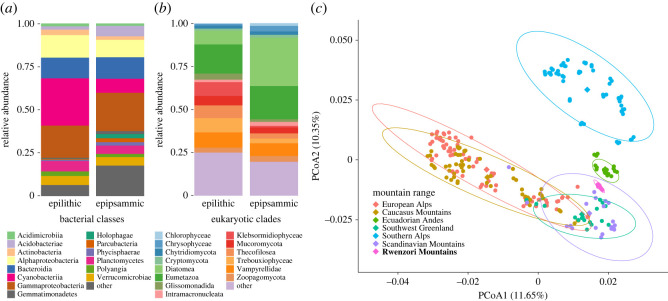
Taxonomic composition of the microbiome in the Mt. Stanley GFS. Relative abundances of bacterial (*a*) and eukaryotic groups (*b*) in epilithic and epipsammic biofilms. Each bar represents an average of three patches from one upstream and one downstream reach. (*c*) Patterns of epipsammic bacterial community composition across mountain ranges illustrated on a PCoA based on Bray–Curtis dissimilarity. Ellipses represent the 95% confidence intervals.

The microbiome of the Mt. Stanley GFS was characterized by a rich photoautotrophic community, which is in line with the elevated benthic algal biomass inferred from chlorophyll-*a*. Our analyses based on the 18S rRNA gene shows that the eukaryotic community of the Mt. Stanley GFS epipsammic and epilithic fractions exhibit a different structure compared to those of GFSs from higher latitudes (PERMANOVA, epipsammic: *F* = 9.21, *p* = 0.001; epilithic: *F* = 16.3, *p* = 0.001; pairwise comparison Adonis tests, *p* < 0.002 for all tests). For instance, we found an increased abundance of diatoms in epipsammic biofilms of the Mt. Stanley GFS (34.2% ± 11.3%) compared to other high-latitude GFSs (approx. 2%)(ANCOM, *W* = 12.4, *p* < 0.01). On the other hand, members from the iconic golden algae *Chrysophyceae* (particularly *Hydrurus*), abundantly occurring in GFSs worldwide (greater than 20%, as the relative abundance of gene copies; [21,34,61,62]), were largely missing from epipsammic biofilms (less than 0.01%) in the Mt. Stanley GFS (*W* = −8.8, *p* < 0.001) (figure 2). Next, while we noted similar values of OTU richness for both the epilithic and epipsammic biofilms (electronic supplementary material, figure S1a, *W* = 14, *p* = 0,57), the structure of their communities significantly differed (PERMANOVA, *F* = 4.07, *p* = 0.002). We found increased relative abundances of *Ochrophyta* in epipsammic biofilms, which includes numerous diatoms (e.g. genera *Achnanthidium, Neidium* and *Cymbopleura*) (figure 2; electronic supplementary material, table S5). By contrast, epilithic biofilms were dominated by *Charophyta* (*W* = 2.8, *p* < 0.03), *Chlorophyta* (*W* = 2.6, *p* < 0.03) and *Cercozoa* (*W* = 2.7, *p* < 0.03), and included groups such as *Klebsormidiophyceae*, *Trebouxiophyceae* and *Vampyrellidae*.

Our analyses also revealed numerous fungi associated with the Mt. Stanley GFS microbiome, including *Mucoromycota*, *Zoopagomyctoa* and *Chytridiomycota* (figure 2*b*; electronic supplementary material, table S5). The occurrence of these fungi suggests a relatively complex microbial food web in the Mt. Stanley GFS, likely sustained to some extent by the elevated primary production. In fact, *Chytridiomycota* are known as algal parasites, thereby inducing a fungal shunt with potential implications for carbon cycling [63,64]. It should be noted, however, that *Chytridiomycota* occurred at lower relative abundances than in other higher-latitude GFSs, whereas *Mucoromycota* and *Zoopagomyctoa* were apparently more abundant (electronic supplementary material, table S5). The latter can be parasites, pathogens or saprobes [65]. The notion of a well-developed microbial food web is further corroborated by the presence of predatory amoebae, notably *Vampyrellidae* (epipsammic: 9% ± 1.2%; epilithic: 10.3% ± 5.3%). *Vampyrellidae* have been reported to predate on eukaryotic cells, including green algae and diatoms [66] and to abundantly occur in periglacial habitats on Mt. Kilimanjaro [8].

Further, we noted similar values of bacterial ASV richness in epilithic and epipsammic biofilms as compared to other GFSs from Caucasus and New Zealand (electronic supplementary material, figure S1b [34]). However, the Mt. Stanley GFS showed differing patterns in bacterial (16S rRNA gene) community structure from other GFSs around the world [21,34,67] for both of the sampled habitat types (figure 2*c*; PERMANOVA; epipsammic: *F* = 14.53, *p* = 0.001; epilithic: *F* = 9.2, *p* = 0.001; pairwise comparison Adonis, *p* < 0.002 for all tests). It is worth noting that these studies used the same primers as the present dataset, which made the comparison possible and reliable. At the genus level, *Polaromonas* (*W* = −5.8, *p* < 0.001), *Rhodoferax* (*W* = −6.4, *p* < 0.001), *Methylotenera* (*W* = −7.8, *p* < 0.001) and *Nitrospira* (*W* = −1.7, *p* < 0.001)*,* commonly reported to dominate in terms of prevalence and abundance in higher-latitude GFSs [34,68], were present at low relative abundance in the Mt. Stanley GFS (electronic supplementary material, table S5). Instead, we found increased abundances of members from genera *Calothrix*, Ellin6067 and *Rhizobacter* in epipsammic biofilms (electronic supplementary material, table S5), whereas *Fimbriiglobus, Ferruginibacter* and members from the family *Leptolyngbyaceae* occurred at elevated relative abundances in the epilithic biofilms (electronic supplementary material, table S3). The fact that *Calothrix* and *Leptolyngbyaceae* were relatively abundant in the Mt. Stanley GFS (as compared to other high-latitude GFS) is not unexpected given that these cyanobacteria have been repeatedly reported from the cryosphere (e.g. cryoconites) [8,69,70].

Further exploring the Mt. Stanley GFS bacterial diversity and community structure, we found greater values of ASV richness in the epipsammic compared to epilithic biofilms (electronic supplementary material, figure S1b; *W* = 2, *p* = 0.01), as previously observed in GFS from higher latitudes [34]. In addition, we noted significant differences in bacterial community composition between epipsammic and epilithic biofilms (PERMANOVA, *F* = 6.05, *p* = 0.006). This finding is in line with a recent report of GFSs from New Zealand and the Russian Caucasus [34], and highlights the relevance of sedimentary habitat heterogeneity for microbial communities. In the Mt. Stanley GFS, both habitats were composed of similar taxa, yet with differing relative abundances. Epipsammic biofilms were dominated by members of the class *Gammaproteobacteria*, *Bacteroidiia*, *Alphaproteobacteria* and *Cyanobacteria*, while *Cyanobacteria*, *Gammaproteobacteria* and *Alphaproteobacteria* dominated epilithic biofilms (figure 2*a*; electronic supplementary material, table S5).

Overall, these results indicate that the microbiome structure of the Mt. Stanley GFS differs from other GFSs at higher latitudes. We tentatively attribute these differences to the environment of the Mt. Stanley GFS, characterized by elevated physical stability, which facilitates primary production and a relatively complex microbial food web.

### 3.3. Metagenomic insights into the functioning of the Mt. Stanley GFS microbiome

Metagenomic sequencing, assembly, and binning resulted in 682 non-redundant, medium- to high-quality MAGs, including 327 high-quality prokaryotic MAGs with completeness higher than 90% and contamination lower than 5% [71] (electronic supplementary material, table S3). Among these, only six belonged to Archaea, namely *Methanomicrobiales*, *Nitrososphaerales* and *Methanotrichales*. The most abundant bacterial MAGs belonged to *Gammaproteobacteria*, *Alphaproteobacteria*, *Bacteroidia* and *Verrucomicrobiae*, consistent with our results from 16S rRNA gene analysis and an earlier report from other GFSs [34]. We note that the MAGs accounted for 46.2% ± 7.3% of the reads per samples. This number is higher or similar than those observed in other datasets, e.g. Tara Ocean metagenomes (6.8% [72]) or polar metagenomes (43.3% [73]). Furthermore, we calculated the relative abundance of the MAGs as estimated by coverage and found that while there were differences compared to other measures of taxonomic composition (e.g. 16S rRNA amplicon and read-based taxonomy, electronic supplementary material, figure S2), the global trends were conserved with similar abundant classes such as *Gammaproteobacteria*, *Alphaproteobacteria*, and *Bacteroidia*. Out of the 682 MAGs, 1, 1, 11 and 89 belonged to a new class, order, family and genus, respectively, when comparing with the GTDB database [47]. However, none of them belonged to the same species (as determined by 95%> ANI [71]) as other published GFSs MAGs obtained from the Southern Alps and Caucasus Mountains [34]. Yet, we note that 9 MAGS belonged to the same species as MAGs obtained from Tibetan glaciers [53]. These MAGs belonged to the classes *Acidobacteriae*, *Actinomycetia*, *Bacteroidia* (2), *Alphaproteobacteria* (2) and *Gammaproteobacteria* (3), and accounted for less than 2.8% of the community. Overall, these results confirm the uniqueness of the microbial community in this equatorial cryospheric environment.

**Figure 3.**
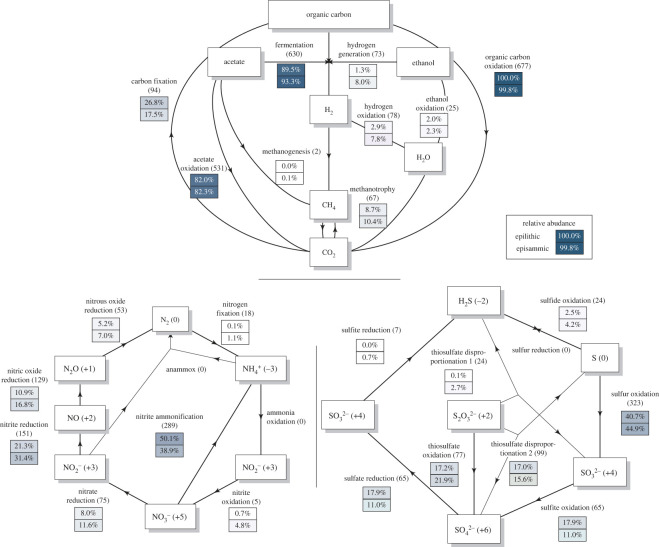
Abundance of organisms (expressed in percentages) involved in different carbon, nitrogen and sulfur cycling in epilithic and epipsammic communities of the GFS. Each sub-pathway is indicated as a step with the corresponding percentage of genomes encoding the respective genes. The flow grams were created using a modified script from METABOLIC [50].

Consistent with the autotrophic character of the Mt. Stanley GFS, we found that ca. 30% and 17.5% of the MAGs from epilithic and epipsammic biofilms, respectively, had carbon (CO_2_) fixation genes (figure 3), reflecting the higher contribution of *Cyanobacteria* to the epilithic microbiome. Furthermore, nearly all (greater than 99.8%) MAGs were able to oxidize organic carbon, and 80% of the MAGs had the potential for aerobic fermentation and acetate oxidation while less than 10% of the MAGs were associated with anaerobic carbon metabolisms (e.g. methanogenesis) (figure 3), which reflects the well-oxygenated environment of the Mt. Stanley GFS.

Next, we screened MAGs for pathways involved in the nitrogen cycle. Interestingly, nitrogen fixation, ammonia oxidation, nitrite oxidation and anammox metabolism were nearly absent in the Mt. Stanley GFS microbiome (figure 3). This is consistent with previous observations from GFSs in New Zealand and the Russian Caucasus [34]. However, dissimilatory nitrate reduction to ammonium (DNRA, or nitrite ammonification) and denitrification were found in up to 50% of the MAGs. Indeed, MAGs with DNRA were more abundant in epilithic biofilms, while MAGs with denitrification potential were more abundant in epipsammic biofilms (figure 3). As suggested previously [34], DNRA could enhance nitrogen recycling within epilithic biofilms through ammonia assimilation by eukaryotic algae and *Cyanobacteria*. Our results suggest a similar process, though probably more driven by *Cyanobacteria*, as, unlike in other GFSs, *Chrysophytes* (e.g. *Hydrurus* spp.) were not abundant in the Mt. Stanley GFS (figure 2).

We further screened the MAGs for pathways involved in sulfur metabolism as glacial bedrock is a potential source of minerals that chemolithotrophic microorganisms are known to use [74]. Despite the low sulfate concentration (1.4–1.5 µg l^−1^) in the Mt. Stanley GFS water, we detected a relatively high proportion of potential sulfur oxidation (320 MAGs, 40–44.7% coverage), sulfite oxidation (64 MAGs, 10.1–17.9% coverage), sulfate reduction (64 MAGs, 10.9–17.9% coverage), thiosulfate oxidation (77 MAGs, 17.2–21.9% coverage) and thiosulfate disproportionation 1 (98 MAGs, 15.6–16.7% coverage). Strikingly, the main contributors to sulfate reduction were the *Cyanobacteria,* which accounted for 64.4% and 35% of the coverage of the genes involved in this process in the epilithic and epipsammic biofilms, respectively. *Cyanobacteria* either reduce sulfate for protein and coenzyme synthesis or sulfolipid biosynthesis [75]. The latest is synthesized with the genes *sqdB* and *sqdX*, consistently present in our cyanobacteria MAGs (*n* = 10). This lipid, typical for most photosynthetic organisms, is usually degraded during sulfur starvation to yield sulfur for the protein synthesis [76]. This finding suggests that *Cyanobacteria* in the Mt. Stanley GFS are subjected to prolonged periods of low sulfate concentrations.

**Figure 4.**
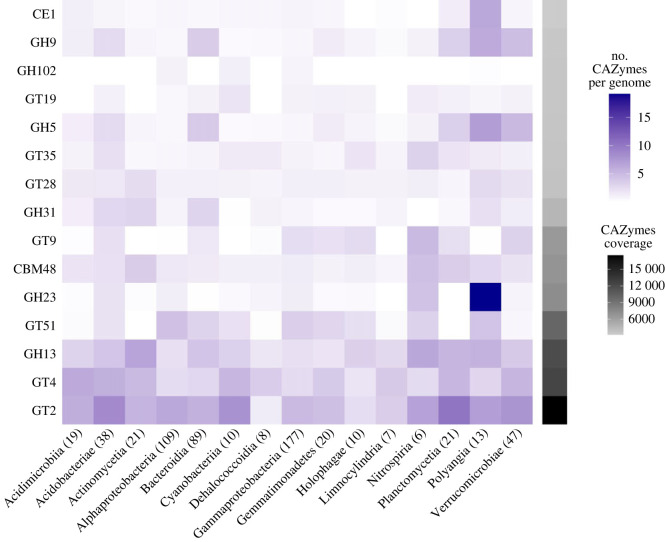
Heatmap representing the number of different CAZymes genes per prokaryotic MAGs (purple colour gradient) in the 15 most abundant taxonomic classes. For all CAZymes categories, the sum of the coverage of each gene is indicated on the right (black colour gradient). The numbers in parenthesis correspond to the number of MAGs per classes. The CAZYmes categories are CE (carbohydrate esterases), GH (glycoside hydrolases), GT (glycosyltransferases) and CBM (carbohydrate-binding modules).

Given the abundant primary producers in the Mt. Stanley GFS, we specifically screened genes and proteins degrading polysaccharides, the most abundant components of the algal exudates [77]. First, we looked at the most abundant CAZymes families present and determined which taxa were most probably responsible for the degradation of the various polysaccharides (figure 4). Overall, GT2 (Glycosyltransferases), GT4 and GH13 (Glycoside Hydrolase) were the most abundant and prevalent CAZymes across all MAGs. GTs catalyze the synthesis of glycosidic bonds by harnessing activated sugar donors to many acceptor substrates and are thus essential to energy generation. GT2 (i.e. cellulose synthase, chitin synthase, mannosyltransferase) and GT4 (i.e. sucrose synthase, sucrose-phosphate synthase, α-glucosyltransferase) are known to predominate in many organisms, especially bacteria [78,79]. Similarly, GH13, which is part of the glycoside hydrolase family, also known as α-amylase, is among the largest glycoside hydrolase family [80]; therefore, its abundance and prevalence in all MAGs are not unexpected.

Focusing on the other abundant CAZymes, we noted the elevated abundance (greater than 15 copies per MAG) of GH23, comprising peptidoglycan lyase and chitinase, in the *Polyangia* class. This is remarkable as Polyangiaceae are known for their predatory lifestyle, able to lyse prokaryotic and eukaryotic cells, including algae and yeast [81]. Furthermore, we found high copy numbers of GH31 (i.e. α-glucosidase, α-galactosidase, α-mannosidase), GH5 (i.e. endo-β-1,4-glucanase, β-glucosidase, endo-β-1,4-xylanase) and GH9 (i.e. endoglucanase, endo-β-1,3(4)-glucanase, lichenase) associated with MAGs from *Bacteroidia* and *Polyangia*. Interestingly, GH5 includes β-glucosidase, of which we measured high process rates in the Mt. Stanley GFS (table 1). We, therefore, infer that the elevated β-glucosidase activity is likely attributable to representatives from the *Bacteroidia* class, which is the third most abundant group in the Mt. Stanley GFS as is already the case in other GFSs (figure 2; electronic supplementary material, figure S3) [21]. Furthermore, the GH5 and GH16 have been linked to the degradation of chrysolaminarin, one of the main polysaccharides produced by diatoms [82].

**Figure 5.**
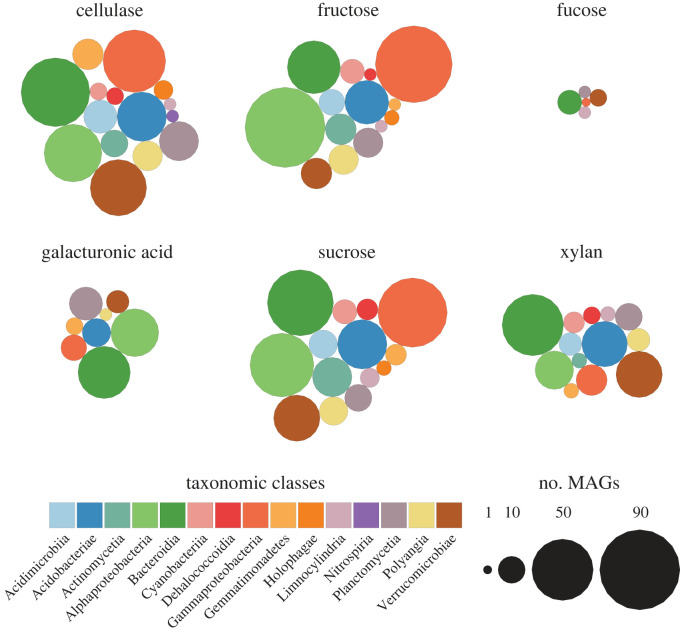
Abundance of prokaryotic MAGs able to degrade polysaccharides coloured by taxa. The size of the circles is representative of the number of MAGs of a specific class possessing the genes known to be involved in the degradation of the selected compounds (i.e. cellulase, fructose, fucose, galacturonic acid, sucrose and xylan).

Finally, motivated by our findings on the EEAs, we focused on specific pathways involved in the degradation of cellulase, fucose, fructose, lactose, mannose, sucrose and xylose—all of them major constituents of algal polysaccharides [83,84] (figure 5). Surprisingly, 58% (395) of the bacterial MAGs, foremost *Bacteroidia* (72%, 64) and *Verrucomicrobiae* (91.5%, 43), possessed cellulase genes. The fact that these two classes also contributed the most to the xylanase composition with 57.3% (51) and 61.7% (29) of MAGs, respectively, suggests that they are the main degraders of polysaccharides deriving from green algae and mosses in the Mt. Stanley GFS. Following this, we observed that 122 MAGs (37.3%), mainly belonging to *Alphaproteobacteria* (28.4%; 31) and *Bacteroidia* (41.6%; 37), possess genes involved in the degradation of galacturonic acid, which is a main component of pectin also found in green algae. We also note that, consistent with the low abundance of *Chrysophytes* (e.g. golden and brown algae), very few MAGs possessed genes involved in fucose degradation, the main component of fucoidan, a polysaccharide found in these algae. Overall, these results show that the bacterial community is well adapted to the degradation of the polysaccharides typically associated with the specific algae present in the Mt. Stanley GFS.

## 4. Conclusion

Our findings set apart the Mt. Stanley GFS from other GFSs at higher latitudes. Both the tropical climate and small size of the Mt. Stanley Glacier collectively impart low temporal variations and magnitudes on runoff which stabilizes the GFS channel. Environmental stability over extended time periods promotes the growth of benthic primary producers, with marked impacts on the microbiome structure and function of the Mt. Stanley GFS, even with potential consequences for biogeochemical cycles. Our findings provide first insights into a now rapidly vanishing GFS, likely typical for other African cryospheric systems. At the same time, the Mt. Stanley GFS serves as a ‘crystal ball' for high-latitude GFSs, which are predicted to experience a less harsh and more stable environment as their glaciers shrink [14]. We may therefore expect the future GFS microbiome to become more like the Mt. Stanley GFS microbiome, with autotrophic-heterotrophic processes gaining greater relevance over chemolithoautotrophy. Collectively, our findings underline the need to conduct thorough assessments of the biological diversity of further African GFSs, which are putative hotspots for unique microbial biodiversity.

## Data Availability

Raw sequence data have been deposited in the Sequence Read Archive (SRR20627829-SRR20627866) with NCBI BioProject accession no. PRJNA781406. The MAGs were deposited in with the accessions JANXLO000000000-JANYAF000000000 and JANYAG000000000-JANYLS000000000 using the same NCBI BioProject. The data are provided in electronic supplementary material [85].
